# Annual and Other Meetings

**Published:** 1919-06-21

**Authors:** 


### Annual and Other Meetings.

### Great Ormond Street Hospital.

LORD ABERDARE presided over the annual Court of Governors. Last year 2,567 in-patients and 26,899 new out-patients, with 80,996 attendances, were treated. Notwithstanding a decrease of £284 in the annual subscriptions, there was an increase of £1,658 in income as compared with 1917. Expenditure had increased by £3,539 in 1918 in spite of the most rigid economy. The year under review started with a debt of £4,500 to the bankers, which, however, has been completely cleared off by a grant from the King's Fund and the payment of a legacy from the late Miss L. S. Scott.

### Royal Eye Hospital.

LORD SOUTHWARK occupied the chair at the annual general meeting. Last year 514 new in-patients and 21,848 new out-patients, with 56,513 attendances, were cared for. Since the outbreak of hostilities 305 wounded soldiers and sailors had occupied the eighteen beds. Income in 1918 amounted to £7,789, as against £7,849 a year previously, while expenditure totalled £6,811 as compared with £5,963 in 1917. The need for new annual subscriptions and generous donations was very urgent, and it was satisfactory to note that the former showed an increase of over £40 last year.

### Belgrave Hospital for Children.

AT the annual Court of Governors it was reported that 560 patients, 132 of whom were under one year of age, were admitted to the wards last year. New out-patients numbered 9,419, and their attendances totalled 33,711. Expenditure amounted to £6,196, as compared with £5,536 in 1917, while income decreased from £5,586 in that year to £5,038 during the year under review. The Prince of Wales, who has contributed £50 to the funds, has been elected a Life Governor. The Ladies' Association have rendered valuable assistance during the year, the Work Branch having made 125 garments of various kinds for the use of the patients.

### Victoria Hospital, Chelsea.

AT the annual meeting of Governors it was announced that the number of patients admitted to the institution last year was 1,088—a decrease of 101 as compared with 1917. There was also a falling-off in out-patients, which was probably largely due to the work done at the L.C.C. School Clinics, where many minor ailments were now treated. The number of new out-patients was 12,228, and their attendances 57,754. Last year was a successful one financially, the ordinary income having been £611 in excess of 1917. In addition legacies to the amount of over £2,000 had been received. Expenditure showed an increase of £727. Heavy calls upon the finances of the hospital were anticipated in the immediate future. The improvement in the method of heating by the installation of a modern system of central steam heating, involving an expenditure of about £3,000, could not long be delayed. There was also the need for increasing the salaries and wages of the staff and reducing their hours of duty.

